# Fatal Acute Airway Obstruction During Bronchoscopy-Guided Percutaneous Tracheostomy: An Analysis of a New Complication

**DOI:** 10.7759/cureus.43593

**Published:** 2023-08-16

**Authors:** Shailja Kataria, Diego Hanssen, Mohammed Kassem, Sandeep Kataria, Daniel T Farkas

**Affiliations:** 1 General Surgery, BronxCare Health System, Bronx, USA; 2 Surgery, BronxCare Health System, Bronx, USA; 3 Anesthesiology, BronxCare Health System, Bronx, USA

**Keywords:** loss of airway, thick secretions, obstructed endotracheal tube, bronchoscopy, percutaneous tracheostomy

## Abstract

Percutaneous tracheostomy (PT) is a commonly performed procedure in ICUs as a safe and cost-effective alternative to surgical tracheostomy (ST). Bronchoscopy is frequently used during PT for real-time confirmation of needle placement and tube positioning. We present a case of a 42-year-old female with a complex medical history who underwent PT and experienced acute airway loss due to endotracheal tube obstruction caused by accumulated secretions. To prevent such complications, vigilance regarding airway obstruction, cautious bronchoscope advancement, avoiding endotracheal tube puncture, and readiness to abort the procedure and replace the tube are crucial.

## Introduction

Percutaneous tracheostomy (PT) has emerged as a safe and cost-effective alternative to surgical tracheostomy (ST) in ICUs. There is extensive evidence to support PT's comparable safety profile when performed by trained clinicians. PT's bedside execution in the ICU offers convenience and eliminates operating room and anesthesia charges, resulting in significant cost savings [1]. Bronchoscopy is widely used while performing PT [2] and its proposed advantages include real-time confirmation of needle placement, ensuring midline positioning of the needle, verifying tube placement, and preventing injury to the posterior tracheal wall. However, the evidence supporting these benefits is limited and retrospective, and not strong enough to clearly recommend bronchoscopy as a routine part of PT [3]. Nonetheless, in a recent randomized controlled trial that aimed to examine the safety of PT using bronchoscopy [4], the overall complication rates of PT compare favorably with those of ST. The heterogeneity of PT techniques adds to the challenge of interpreting the published data and meta-analyses. Mortality rates of tracheostomy are less than 1% based on several large series [1]. Immediate procedure-related complications are rare and include bleeding and loss of airway due to accidental extubation and tracheostomy in the false passage [5]. In this report, we discuss a new complication that has not been reported previously in the literature. We observed acute loss of airway due to sudden obstruction of the lumen of the endotracheal tube during PT in a female patient.

## Case presentation

The patient was a 42-year-old female with a complex medical history, including HIV/AIDS, polysubstance use disorder, and bipolar disorder. The patient was admitted to the hospital with pneumonia and she had a history of non-compliance with highly active antiretroviral therapy (HAART). She underwent bronchoscopy with bronchiolar lavage and was diagnosed with pneumocystis pneumonia (PCP). She was treated with sulfamethoxazole-trimethoprim while in the hospital, but she later left against medical advice. The patient returned to the hospital several days later and within several days developed worsening respiratory failure. She had tachypnea, with increasing oxygen requirements, and intubation was required. She underwent a repeat bronchoscopy, which still showed the presence of PCP.

Due to the patient's prolonged need for mechanical ventilation, a surgical consultation was requested on the eighth day of intubation to discuss the possibility of performing PT, as per the family's preference for shifting her to a nursing home. Though the patient's ventilatory requirements were not optimal, she was felt to be ready for PT. She was on FiO_2_ of 60% with a positive end-expiratory pressure (PEEP) of 5. She had elevated PCO_2_ of 57 but this had been persistent for most of her hospitalization and had improved compared to the day before. Similarly, she had an elevated peak pressure of 49 cmH_2_O. This was slightly increased from 45 the day before. She was not coagulopathic, with an INR of 1.14 and a platelet count of 188. As such, we proceeded with the PT procedure.

During the procedure, a 5 mm fiberoptic bronchoscope was inserted through the previously placed size 7 mm endotracheal tube. Initially, the distal end of the tube was not easily identified, but a narrow opening was eventually found, obstructed by thick crystalized secretions. While attempting to reposition the endotracheal tube to ensure a suitable location for the tracheostomy, the patient experienced a sudden drop in oxygen saturation. Promptly, manual ventilation with an Ambu bag was initiated. Unfortunately, the patient's saturation further decreased, accompanied by bradycardia and subsequent cardiac arrest. The patient had a "do not resuscitate" (DNR) status, and while no resuscitation was started, the ICU team immediately contacted the patient's family to confirm their wishes.

While awaiting the family’s response, we used the bronchoscope to confirm that the endotracheal tube was still in the airway. The bronchoscope was passed down the endotracheal tube, confirming that it was still within the airway. However, the secretions coating the inner side of the tube were observed to be flaking off and going distally, causing obstruction of the airway. Attempts to suction the secretions using the bronchoscope were unsuccessful.

**Figure 1:**
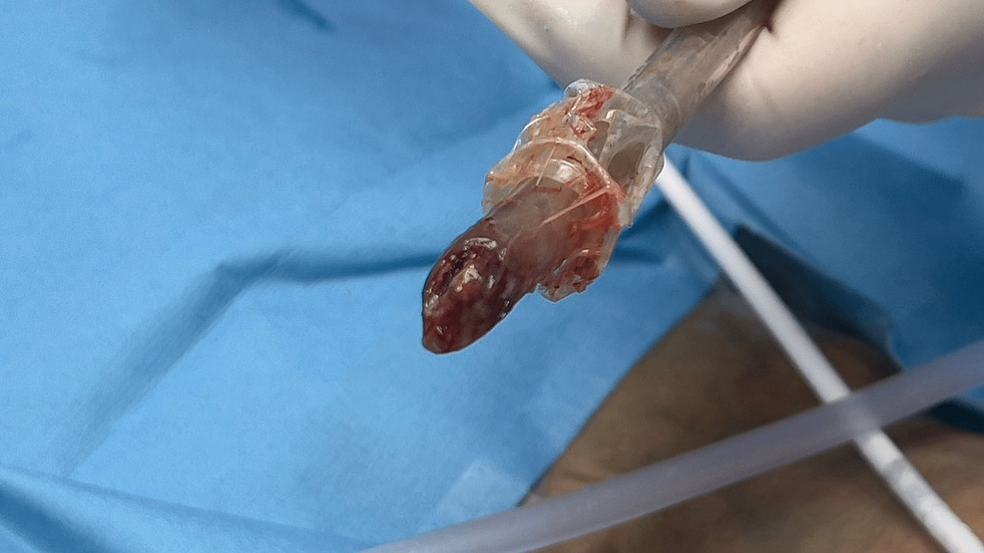
Thick coating of dried secretions causing obstruction at the distal end of removed endotracheal tube

At this point, the family confirmed their preference for a DNR status, allowing the patient to pass away peacefully. Subsequently, the endotracheal tube was removed (Figure 1). It revealed a thick coating of dried secretions causing obstruction at the tip of the endotracheal tube.

## Discussion

Endotracheal tube obstruction is a known complication associated with mechanical ventilation, although its precise incidence remains uncertain. The clinical presentation typically manifests as increased airway resistance, difficulty in passing suction catheters, and changes in pressure and flow tracings on the ventilator. This presentation tends to progress gradually, leading to early intervention through endotracheal tube exchange. In this case report, we describe a severe instance of endotracheal tube obstruction encountered during PT, which resulted in a fatal outcome.

Endotracheal tube obstruction can arise from various factors, including tube kinking, patient biting, cuff hyperinflation and herniation, foreign bodies, or mucus plugging. Mucus plugging has been observed, particularly in patients with severe asthma who require mechanical ventilation. However, to the best of our knowledge, peri-procedural endotracheal tube obstruction during the bronchoscopy examination segment of tracheostomy has not been previously reported.

We hypothesize that the acute decompensation in our case resulted from complete obstruction of the airway due to the dislodgement of accumulated inspissated secretions. This observation was made during the bronchoscopy procedure. We observed the presence of inspissated secretions along the inner wall of the endotracheal tube, forming a sheet-like accumulation. These secretions were actively flaking off from the tube, resulting in their accumulation and subsequent obstruction at the distal end of the tube (Figure 1). Consequently, it became challenging to identify the end of the tube and visualize the tracheal lumen. This may have been caused partially due to the bronchoscopy, but an additional complicating factor was our use of a finder needle for the tracheostomy. The needle may have punctured the endotracheal tube, and introduced some air around the secretions, making them easier to flake off from the inside of the tube. Our patient also had PCP, and some case reports have indicated an association between PCP pneumonia and the presence of thick, adherent secretions [6].

To mitigate the risk of endotracheal tube obstruction during similar procedures, we propose the following measures: firstly, it is crucial to maintain a high level of suspicion for possible airway obstruction. This involves closely monitoring the patient for signs such as increasing PCO_2_ and rising airway pressures, especially when there is no other apparent explanation. Secondly, when advancing the bronchoscope, it is essential to exercise caution and remain vigilant, particularly if inspissated secretions are identified within the tube. Utmost care should be taken to avoid puncturing the endotracheal tube with the finder needle, even though it may not be part of the planned procedure. This aspect requires specific attention, especially when learners/trainees are involved in the procedure. Lastly, it is of paramount importance to have a low threshold for aborting the procedure and replacing the tube before attempting it again. By promptly recognizing thick secretions in the endotracheal tube and taking necessary action, we can effectively reduce the risk of endotracheal tube obstruction and ensure patient safety.

Subsequent to the case in this report, we encountered a similar case where partial obstruction of the end of the endotracheal tube was identified. The procedure was aborted, and the bronchoscope was removed. The endotracheal tube was replaced using a laryngoscope, and the original tube had a large mucus plug. Once the new tube was in, we proceeded with PT under bronchoscopic guidance, and the patient tolerated it without any issues.

## Conclusions

We described a new complication that we observed during a PT procedure: sudden endotracheal tube obstruction in a previously partially obstructed tube. To prevent this, it is recommended to exercise increased vigilance and avoid needle trauma to the endotracheal tube when encountering significant inspissated secretions. In selected cases, consideration should be given to pre-procedure replacement with a new endotracheal tube.

## References

[1] Al-Shathri Z, Susanto I (2018). Percutaneous tracheostomy. Semin Respir Crit Care Med.

[2] Kluge S, Baumann HJ, Maier C, Klose H, Meyer A, Nierhaus A, Kreymann G (2008). Tracheostomy in the intensive care unit: a nationwide survey. Anesth Analg.

[3] Raimondi N, Vial MR, Calleja J (2017). Evidence-based guidelines for the use of tracheostomy in critically ill patients. J Crit Care.

[4] Añón JM, Arellano MS, Pérez-Márquez M (2021). The role of routine FIBERoptic bronchoscopy monitoring during percutaneous dilatational TRACHeostomy (FIBERTRACH): a study protocol for a randomized, controlled clinical trial. Trials.

[5] Putensen C, Theuerkauf N, Guenther U, Vargas M, Pelosi P (2014). Percutaneous and surgical tracheostomy in critically ill adult patients: a meta-analysis. Crit Care.

[6] Siegel RE (1987). Occlusion of the endotracheal tube. Chest.

